# A case of acute functional hemispherotomy in a young woman with hemimegalencephaly and super-refractory status epilepticus

**DOI:** 10.1016/j.ebr.2024.100728

**Published:** 2024-11-17

**Authors:** Kjell Heuser, Louis Romundstad, Jugoslav Ivanovic, Arild Egge, Erik Sætre, Kristin Alfstad, Line Sveberg, Line Bedos Ulvin, Erik Taubøll

**Affiliations:** a Department of Neurology, Oslo University Hospital, Oslo, Norway; b Department of Anesthesia and Intensive Care Medicine, Division of Emergencies and Critical Care, Oslo University Hospital, Oslo, Norway; c Lovisenberg Diaconal University College, Oslo, Norway; d Department of Neurosurgery, Division of Stereotactic and Functional Neurosurgery, Oslo University Hospital, Rikshospitalet, Oslo, Norway; e National Center for Epilepsy, Member of the ERN EpiCARE, Oslo University Hospital, Oslo, Norway; f Section for Clinical Neurophysiology, Department of Neurology, Oslo University Hospital, Oslo, Norway; g Faculty of Medicine, University of Oslo, Oslo, Norway

**Keywords:** Epilepsy, Status epilepticus, Super-refractory status epilepticus, Epilepsy surgery, Hemimegalencephaly, Functional hemispherotomy

## Abstract

Status epilepticus (SE) is a critical medical emergency that demands immediate and effective intervention. We report a unique case involving a 21-year-old woman with left hemimegalencephaly who was hospitalized for super-refractory status epilepticus (SRSE) that persisted for 8 weeks. Despite extensive treatment efforts including multiple anti-seizure medications, anesthetics, high-dose methylprednisolone, anakinra, magnesium infusion, and a ketogenic diet, her condition remained unchanged. Ultimately, a left functional hemispherotomy was performed, informed by MRI findings and EEG results showing predominantly left-sided epileptic activity. This decision was made collaboratively when all other therapeutic options had been exhausted. Postoperatively, the patient recovered with manageable neurological deficits and a satisfactory quality of life. To our knowledge, this is the first reported case of acute functional hemispherotomy in an adult with hemimegalencephaly and SRSE.

## Highlights

• Surgical treatment of super-refractory status epilepticus is rarely performed.
• In focal status epilepticus surgical treatment should be considered early.
• Surgery is a treatment option in focal status epilepticus in adult hemimegalencephaly.

## 1 Introduction

Status epilepticus (SE) is a severe neurological emergency with high morbidity and mortality [1], [2]. It occurs when mechanisms fail to terminate seizures, leading to prolonged epileptiform brain activity [1]. This can cause significant brain damage and systemic complications [2]. Rapid cessation of seizures is the primary treatment goal [3], [4]. Refractory SE, not controlled by initial treatments, requires deep sedation or general anesthesia [3], [4]. Some cases progress into super-refractory SE (SRSE), defined as persisting SE despite 24-hour treatment or recurring upon weaning from anesthesia [5]. There are no standardized treatment protocols for SRSE, though empirical guidelines exist [4], [5]. Surgical intervention for SRSE is rare, with limited cases in the literature [6]. Here, we report the first surgical treatment for SRSE in an adult with hemimegalencephaly (HME).

## 2 Case report

### 2.1 Medical history

We present the case of a 21-year-old woman who was diagnosed with epilepsy at the age of 4, with a family history of valvular heart failure. In childhood her language development was slow but complete. Her epilepsy initially seemed well-controlled with valproate (VPA). However, when VPA tapering was attempted at age 10, her epilepsy became more refractory. An MRI performed at age 9 was described normal. Over the next two years, various anti-seizure medications (ASMs) were administered with partial success. Her condition deteriorated further at age 13, prompting the introduction of additional ASMs, including topiramate (TPM), levetiracetam (LEV), lamotrigine (LTG), zonisamide (ZNS), clobazam, and re-introduction of valproate. Despite a diagnosis of mild mental retardation, she was able to attend school, live at home, and maintain relatively normal physical functioning. EEG results consistently showed focal epileptic activity in the left hemisphere. Socially, she remained functional and between age 13–19 she was active with singing, dancing, and hiking. Her motor skills from right side extremities were described as slightly suboptimal.

From age 19 she experienced a serious worsening of her epilepsy, which lead to numerous hospitalizations in the following two years. This progression was initiated by an acute episode of loss of consciousness probably with cardiovascular arrest. Genetic examinations during the years were negative. At the time of progression, she lived in a care home with staff, participated in daily activities, with no gait problems.

### 2.2 Seizure semiology and diagnostics

Four different types of seizures were observed over the years:

1. Focal onset seizures with impaired awareness and motor symptoms (eye opening and deviation to the right, and extension/shaking of the right arm).

2. Focal onset seizures with impaired awareness and nonmotor/autonomic features (pupillary dilation).

3. Probable psychogenic non-epileptic seizures (PNES).

4. Episodes of bradycardia and arrhythmia that we were not able to classify properly, these could be focal onset seizures without impaired awareness and with autonomic features, or unclassified seizures with unknown onset.

The Long-term electroencephalographic monitoring (EEG) showed an ictal pattern originating from the left hemisphere, middle to posterior region, spreading over to the entire left hemisphere. The MRI now showed clear pathologic findings with hemimegaloencephaly in her left hemisphere with extensive migration disorder. Retrospectively the MRI performed when she was 9 years old already showed mild signs of left sided hemimegaloencephaly, but with a clear progression between age 9 and age 19.

### 2.3 Status epilepticus episode

**Fig. 1:**
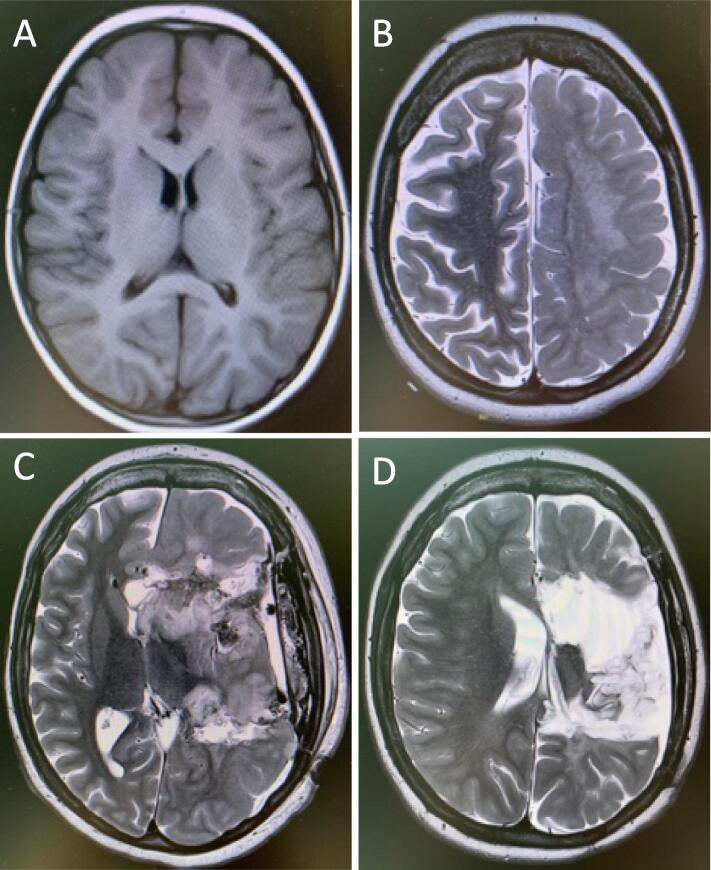
**Cerebral MRI at 4 different time points during the disease course. A)** Age 9: MRI supposed to be normal, however retrospectively one can see signs of hemispheric asymmetry suggesting left-sided hemimegalencephaly. **B)** Age 21: three weeks after SRSE onset: MRI with clear left-sided hemimegaloencephaly. with diffuse confluent T2/Flair hyperintensity in white matter in left fronto-parietal region representing white matter demyelination and gliosis. **C)** MRI 3 weeks after surgery: Status after left-sided functional hemispherotomy. Pronounced edema in the left hemisphere with a midline shift of 11 mm to the right. Mainly signs of vasogenic edema in the basal ganglia on the left side. **D)** MRI 3 months after surgery: Status post left-sided functional hemispherotomy with encephalomalacia and resection defects in the left cerebral hemisphere. Resorption of blood, now with remaining blood products/hemosiderin in the form of scattered small low-signal foci in the resection cavity and linearly along the edge of the resection frontoparietally and temporally. Thickened dura under the craniotomy on the left side and a narrow (2–3 mm) extra-axial residual rim that may contain blood-mixed fluid. Reduction of postoperative parenchymal edema in the left cerebral hemisphere and regression of mass effect.

**Fig. 2:**
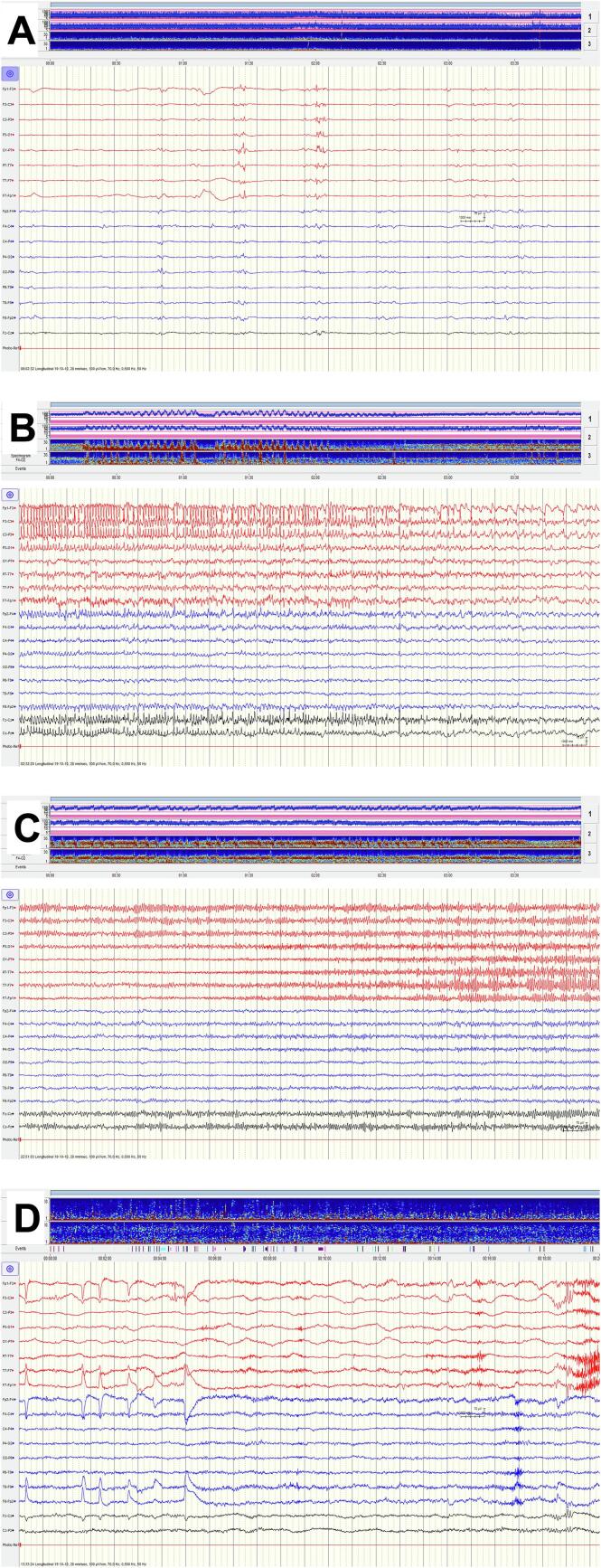
**Excerpts from EEG monitoring during the SRSE episodes and 4 months after functional hemispherotomy. A) EEG 2 weeks after SRSE onset under deep sedation.** Longitudinal montage, vertical lines indicate 1-second intervals. Top panel showing the corresponding amplitude-integrated EEGs (F3-P3/F4-P4) and spectrograms (F3-O1/F4-O2) over a 4 h period. **ASM:** Valproate, Zonisamide, Sultinam. **Sedatives/anesthetics:** Propofol, Thiopental. The EEG shows a burst-suppression pattern with short bursts from 1 to 3 s alternating with suppression periods of 1–3 s. The majority of the bursts may be characterized as highly epileptiform, containing multiple left hemispheric epileptiform discharges. **B) EEG 3.5 weeks after SRSE onset under the second attempt to wake up the patient.** Longitudinal montage, vertical lines indicate 1-second intervals. Top panel showing the corresponding amplitude-integrated EEGs (F3-P3/F4-P4) and spectrograms (F3-O1/F4-O2) over a 4 h period. **ASM:** Sultinam, Valproate, Brivaracetam, Clobazam. **Sedatives/anesthetics:** Dexmedetomidine. **Other:** Meropenem (pneumonia). The EEG shows the end of an electroencephalographic seizure from the left frontocentral area with spreading to the midline and right frontal area. The amplitude-integrated EEGs and spectrograms show repeated seizures (>35) almost without free interval in the first 2 h period (seizures are seen as abrupt increases in amplitude and high frequencies). **C) EEG 5 weeks after SRSE onset under the third weaning attempt.** Longitudinal montage, vertical lines indicate 1-second intervals. Top panel showing the corresponding amplitude-integrated EEGs (F3-P3/F4-P4) and spectrograms (F3-O1/F4-O2) over a 4 h period. **ASM:** Brivaracetam, Clonazepam, Perampanel, Clobazam. **Sedatives/anesthetics:** Propofol. **Other:** The EEG shows the beginning of an electroencephalographic seizure from the left frontocentral area spreading to the entire left hemisphere as well as the midline. The amplitude-integrated EEGs and spectrograms show repeated left hemispheric seizures visible as abrupt increases in amplitude and high frequencies over a 4 h period of time. **D) EEG 4 months after the acute functional hemispherotomy.** Longitudinal montage, vertical lines indicate 1-second intervals. Top panel showing the corresponding spectrograms (F3-O1/F4-O2) over a 20 min period. **ASM:** Brivaracetam, Clobazam, Perampanel. The EEG shows sporadic spikes and polyspikes in the left frontal area combined with left hemispheric slowing. Normal activity in the right hemisphere with an 8 Hz posterior dominant rhythm upon eyes closure. No ictal activity.

At the age of 21 the patient presented with a SRSE lasting for 8 weeks. During this episode 4 unsuccessful attempts were made to wake her up. Other complications during the SE episode were pneumonia (three times), derangement of transaminases and ammonia but not to harmful levels, and critical illness neuropathy. ASM given during the SRSE were valproate, clonazepam, levetiracetam, zonisamide, sultinam, clobazam, and perampanel. Sedatives/ anesthetics applied were propofol, ketamin, midazolam, fentanyl, thiopental, and dexmedetomidine. Other treatments used were high dosage methylprednisolone, anakinra, magnesium infusion, and ketogenic diet. All medications were given in accordance with the general practice for SE treatment in our hospital, in adequate doses and for long enough time [7]. EEG and MR findings performed during and after the SRSE are presented in Fig. 1, Fig. 2 respectively. After 7 weeks in SRSE at a time point where retraction of all active treatment was considered, functional hemispherotomy was performed. Histopathology showed brain tissue with dysmorphic neurons, balloon cells and altered cortical lamination, compatible with focal cortical dysplasia type IIb or hemimegaloencephaly.

### 2.4 Postoperative course

On postoperative day 2, the patient remained unresponsive and developed a fever along with pneumonia. A head CT performed on day 3 revealed edema in the left hemisphere, most probably owing to compromised venous circulation. On day 4, an intracranial pressure (ICP) monitor was placed, which showed normal ICP levels. By day 6, the patient was still unresponsive and unable to move. A nerve conduction study was conducted, diagnosing her with critical illness neuropathy.

On day 7, she opened her eyes for the first time, though she still showed no other signs of movement or interaction. The following day, she made her first eye contact and was able to give a slight squeeze with her left hand. A tracheostomy was performed on day 9. By day 14, attempts to mobilize her, requiring the assistance of four people, were unsuccessful due to instability in her neck and spine.

On postoperative day 21, she smiled for the first time and was able to communicate through head nodding. By day 24, she was decannulated, and by day 26, she could speak without significant difficulty. She then entered a long-term rehabilitation program, which led to substantial clinical improvement.

The patient ultimately experienced a moderate right hemiparesis and hemianopia, which were anticipated as consequences of the surgical procedure. However, she had no speech impairments. Today, three years post-surgery, her epilepsy remains well controlled, she is socially active, and enjoys a relatively good quality of life. Notably, she never again developed SE.

## 3 Discussion

This is the first reported case of acute functional hemispherotomy performed in an adult patient with SRSE due to HME. The literature on surgical treatment of SRSE in adults is limited, though surgery is recommended in multiple international guidelines as a potential treatment option. A recent systematic review and meta-analysis encompassing cases from 1980 to 2023 identified only 157 surgically treated SE patients, with a median age of 12.9 years, 70 % of whom underwent resective surgery [8].

Regarding HME, we found only two previous case reports of surgical treatment in adult patients, both of which were elective [9]. HME is a rare cortical developmental disorder involving the overgrowth of one cerebral hemisphere, affecting approximately 1–3 per 1000 children with epilepsy [10]. These children often exhibit intellectual delay, hemiparesis, and drug-resistant epilepsy. The genetic basis of HME is not fully understood, but de novo somatic mutations in components of the PI3K-AKT3-mTOR pathway, which influence protein creation, cell growth, division, and survival, have been identified in some patients [11].

Typically, children with HME are carefully selected for early surgery following thorough pre-operative examinations using various diagnostic modalities. Functional hemispherotomy, which disconnects the epileptogenic hemisphere from the contralateral (healthy) brain, is often chosen. This procedure usually results in significant improvement in seizures and cognitive dysfunction associated with epilepsy and has a low mortality rate [12], [13]. The perioperative mortality is generally low but worsening of contralateral motor function is mandatory and hydrocephalus may occur. However, most data on outcomes and complications come from elective surgeries in well-prepared patients and families.

In our case, the literature lacked any comparable cases. After all, the patient was relatively functional over many years with reasonable seizure control, and the MR at age 9 only retrospectively revealed early signs of HME. Therfore, surgery was not regarded as indicated until the serious situation with SE emerged. However, MRI at age 19 showed clear worsening compared to the MRI at age 9, which may have served as a prognostic marker. Early surgical intervention when the patient's epilepsy first became refractory might have prevented the onset of SRSE.

After 7 weeks of SRSE and exhaustive medical treatment, the options were either severe impairment or death following the withdrawal of all medical treatment. The alternative was to surgically remove the left epileptic focus via functional hemispherotomy. Typically, a left-sided hemispherotomy results in right-sided hemiparesis/hemiparalysis and hemianopsia, and usually, disconnection of the left (presumably language-dominant) hemisphere leads to aphasia. However, given the patient's pre-existing left hemisphere pathology and left-handedness, it was considered that her right hemisphere could be functionally dominant, which would preserve her speech function post-surgery. This speculation proved correct.

The decision to proceed with left-sided hemispherotomy was made after thorough discussions in the medical team and with the patient’s parents, considering all potential outcomes, from death and severe disability to hemiparalysis/hemianopsia with or without preserved speech function. The surgery was a collective decision with the family.

## 4 Conclusion

This case represents the first documented positive outcome of an adult treated with functional hemispherotomy for SRSE. It highlights critical clinical and ethical considerations for managing similar cases. Early discussions around surgical options, particularly for patients with focal SE, are essential. Involvement of the epilepsy surgery team is crucial to evaluate all therapeutic avenues. Additionally, consulting an ethics advisor can provide valuable assistance, ensuring a balanced approach to decision-making in complex case like this.

## 5 Ethics statement

The patient provided written informed consent for the release of any identifiable personal details and for the publication of this case report along with associated images. The study adheres to the principles outlined in the Declaration of Helsinki and follows the European Medicines Agency's Guidelines on Good Clinical Practice (GCP-Directive). Additionally, the study complies with the Norwegian law and regulatory authority standards.

## CRediT authorship contribution statement

**Kjell Heuser:** Writing – review & editing, Writing – original draft, Validation, Resources, Project administration, Methodology, Investigation, Conceptualization. **Louis Romundstad:** Writing – review & editing, Writing – original draft, Methodology, Conceptualization. **Jugoslav Ivanovic:** Writing – review & editing, Writing – original draft, Methodology, Conceptualization. **Arild Egge:** Writing – review & editing, Writing – original draft. **Erik Sætre:** Writing – review & editing, Writing – original draft. **Kristin Alfstad:** Writing – review & editing, Writing – original draft. **Line Sveberg:** Writing – review & editing, Writing – original draft. **Line Bedos Ulvin:** Writing – review & editing, Writing – original draft, Methodology. **Erik Taubøll:** Writing – review & editing, Writing – original draft.

## Declaration of competing interest

The authors declare that they have no known competing financial interests or personal relationships that could have appeared to influence the work reported in this paper.

## References

[1] Trinka E., Cock H., Hesdorffer D., Rossetti A.O., Scheffer I.E., Shinnar S. (2015). A definition and classification of status epilepticus—report of the ILAE Task Force on Classification of Status Epilepticus. Epilepsia.

[2] Lv R.J., Wang Q., Cui T., Zhu F., Shao X.Q. (2017). Status epilepticus-related etiology, incidence and mortality: a meta-analysis. Epilepsy Res.

[3] Shorvon S., Ferlisi M. (2011). The treatment of super-refractory status epilepticus: a critical review of available therapies and a clinical treatment protocol. Brain.

[4] Heuser K, Olsen KB, Ulvin LB, Gjerstad L, Taubøll E. Modern Treatment of Status Epilepticus in Adults. In: Czuczwar SJ, editor. Epilepsy [Internet]. Brisbane (AU): Exon Publications; 2022. Chapter 5. 35605086

[5] Rai S., Drislane F.W. (2018). Treatment of refractory and super-refractory status epilepticus. Neurotherapeutics.

[6] Basha M.M., Suchdev K., Dhakar M., Kupsky W.J., Mittal S., Shah A.K. (2017). Acute resective surgery for the treatment of refractory status epilepticus. Neurocrit Care.

[7] Heuser K., Horn M., Samsonsen C., Ulvin L.B., Olsen K.B., Power K.N. (2024). 11;144(4). English, Norwegian.

[8] Jha R., Blitz S.E., Chua M.M.J., Warren A.E.L., Lee J.W., Rolston J.D. (2024). Surgical management of status epilepticus: a systematic review. Epilepsia Open.

[9] Liang S., Zhang G., Li Y., Ding C., Yu T., Wang X. (2013). Hemispherectomy in adult patients with severe unilateral epilepsy and hemiplegia. Epilepsy Res.

[10] Di Rocco C., Battaglia D., Pietrini D., Piastra M., Massimi L. (2006). Hemimegalencephaly: clinical implications and surgical treatment. Childs Nerv Syst.

[11] Lee J.H., Huynh M., Silhavy J.L., Kim S., Dixon-Salazar T., Heiberg A. (2012). De novo somatic mutations in components of the PI3K-AKT3-mTOR pathway cause hemimegalencephaly. Nat Genet.

[12] Goel K., Phillips H.W., Chen J.S., Ngo J., Edmonds B., Ha P.X. (2024). Hemispheric epilepsy surgery for hemimegalencephaly: the UCLA experience. Epilepsia.

[13] Schijns O.E.M.G., Delev D., von Lehe M., van Roost D., Rössler K., Theys T. (2024). Functional hemispheric disconnection procedures for chronic epilepsy: history, indications, techniques, complications and current practice in Europe. A consensus statement on behalf of the EANS functional neurosurgery section. Brain Spine.

